# Enhanced pigment content estimation using the Gauss-peak spectra method with thin-layer chromatography for a novel source of natural colorants

**DOI:** 10.1371/journal.pone.0251491

**Published:** 2021-05-12

**Authors:** Natalia Paulina Twardowska

**Affiliations:** Department of Science, Adcote School, Little Ness, Shropshire, United Kingdom; Universite d’Orleans, FRANCE

## Abstract

Alternative pigment sources that are harmless to human health and can be produced in an eco-responsible way are of great research interest. The experiments undertaken in this study were conducted using autumn leaves of *Aesculus hippocastanum* as potential novel colorant sources. This study focused on improving the Gauss-peak spectra method (a less expensive alternative to high-pressure liquid chromatography) in combination with thin-layer chromatography, leading to the development of a new methodology. The collected leaves were stored at two different temperatures: 20°C and −20°C. The data obtained by spectrophotometric scanning of the samples were analyzed using the Gauss-peak spectra method in the R program with three wavelength ranges: 350–750 nm, 390–710 nm, and 400–700 nm. The results were then assessed for statistically significant differences in the estimated concentrations for the different wavelength ranges regarding (1) total pigment, carotenoid, and chlorophyll concentration (two-sample t-test) and (2) concentration of each indicated pigment (two-way analysis of variance). The results were also tested for differences between the estimated concentrations of samples stored under the different conditions. The Gauss-peak spectra results with and without thin-layer chromatography were statistically compared using a paired t-test. The results showed that thin-layer chromatography greatly enhanced the efficiency of the Gauss-peak spectra method for estimating the major and minor pigment composition without generating high additional costs. A wavelength range of 400–700 nm was optimal for all Gauss-peak spectra methods. In conclusion, the proposed method is a more successful, inexpensive alternative to high-pressure liquid chromatography.

## Introduction

Current studies have shown that artificial food dyes used in the food industry negatively affect human health [1–4]. The experiments undertaken in the present study were conducted using *Aesculus hippocastanum* autumn leaves, which are potential colorant sources as they consist of various chlorophylls [5], carotenoids [6], and anthocyanins [7]. Their processing is advantageous as some fallen leaf disposal methods have harmful effects on human health and the environment [8–10].

High-pressure liquid chromatography (HPLC) is a typically used method for pigment analysis; however, it requires complex equipment, careful maintenance, expensive solvents, and advanced operational skills [11, 12]. The Gauss-peak spectra (GPS) method is an inexpensive alternative proposed by Küpper et al. [13, 14] and modified by Thrane et al. [15]. However, one of the concerns in both these studies was the close similarity of carotenoid absorbance spectra creating a challenge for spectral techniques, making it difficult to distinguish between certain pigments when using the GPS method [15]. The aim of the present study was to suggest an effective solution to this issue and propose an optimal wavelength range for spectrophotometric scanning experiments that must be conducted prior to pigment quantification in investigated leaves using the GPS method. The new method described here is a hybrid between chromatographic and photometric analyses based on the addition of a thin-layer chromatography (TLC) step to the GPS method. It allowed for an inexpensive and more reliable estimation of pigments present in *A*. *hippocastanum* autumn leaves.

## Materials and methods

**Fig 1:**
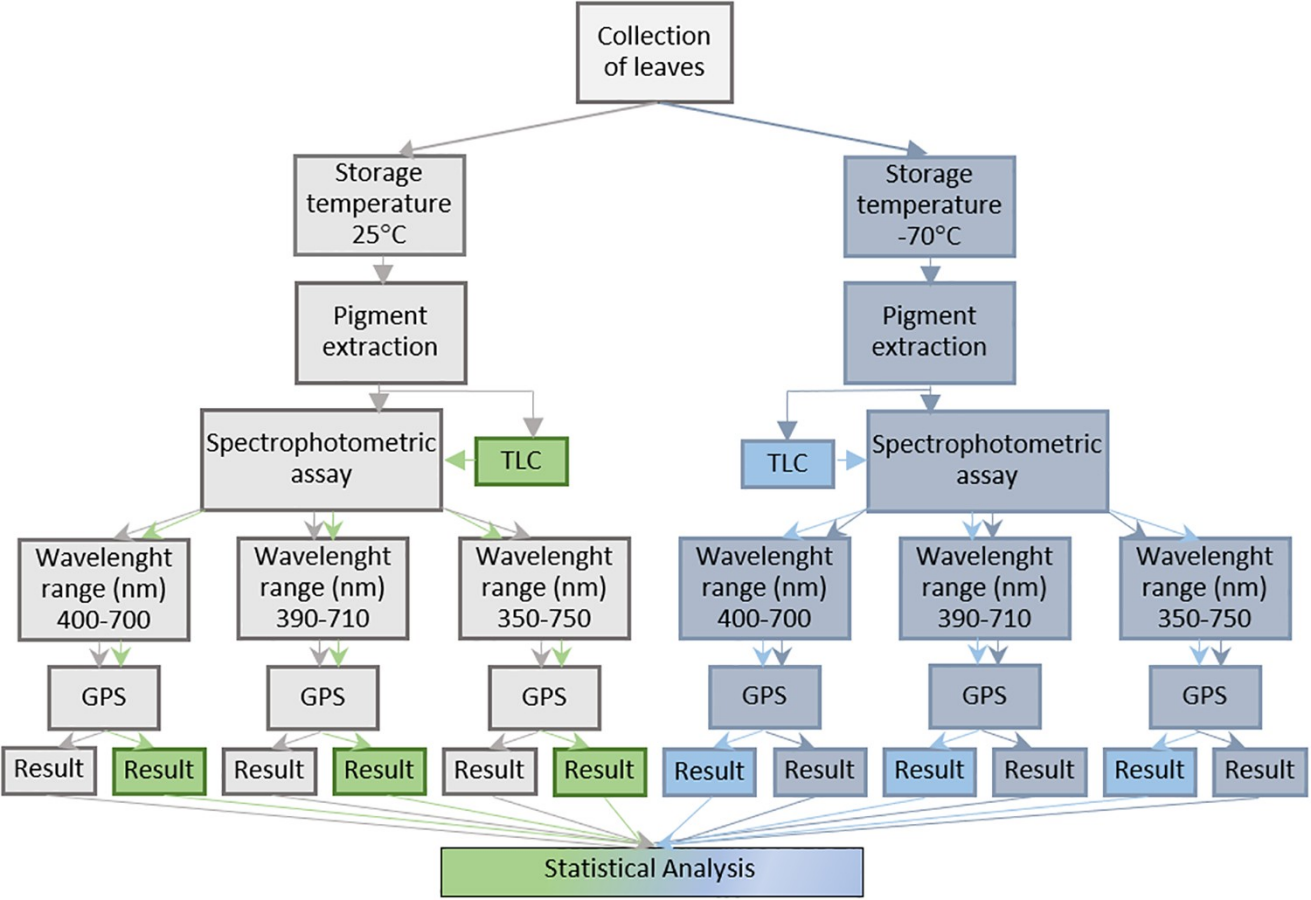
Flow chart of the pigment quantification processes.

The pigment content estimation processes are outlined in Fig 1.

### Leaf collection

*A*. *hippocastanum* leaves were collected from trees in Little Ness, Shrewsbury, Shropshire (52°46′9″ N 2°51′48″ W) in November 2020.

### Leaf preparation

The collected leaves were initially weighed. Next, they were evenly distributed on a flat surface and allowed to dry for 3 h at 20°C in the dark. Then, the leaves were weighed again and left to dry. The steps were repeated until a constant mass of total leaves was achieved after 15 h.

### Storage conditions

The collected leaves were split in half, with one part stored in the dark at room temperature (20°C) and the other part placed in a freezer (−20°C) to prevent pigment degradation [16, 17]. The samples obtained from these leaves were named Group 1 (G1) and Group 2 (G2), respectively. After storage for 10 days, petioles were removed, and the leaves were cut into 5 mm sized pieces for extraction.

### Extraction

Exactly 1 g of leaves from three separate bags of each group (six samples in total) were placed in separate mortar and pestle and supplemented with 15 mL of absolute acetone (acetone for analysis EMSUREACS, ISO, Reag. Ph Eur). Thorough grinding was performed until the venation was white. The acetone was then evaporated, and 5 mL of 96% ethanol (96% Ethanol, EMSURE, Reag. Ph Eur) was used to wash the mortar and pestle and elute the extracted pigments into 15 mL centrifuge tubes. After centrifugation for 10 min at 20°C and 4200 × *g*, the supernatants were collected into clean centrifuge tubes and evaporated until 1 mL of extract was obtained.

### Thin-layer chromatography (TLC)

Silica gel plates (20 cm × 20 cm, TLC Silica gel 60; 20 × 20 cm aluminum sheets, Merck) with a solvent system of 0.8% n-propanol (1-Propanol for analysis EMSUREACS, Reag. Ph Eur) in light petroleum (60–80°C) (petroleum benzene boiling range 60–80°C for analysis EMSURE) (v/v) were used [18, 19]. The entire volume of each sample was transferred onto a silica gel plate with a pipette. The TLC plates were developed at room temperature in the dark for approximately 30 min until the distance of the solvent front reached 17 cm from the origin and were left to dry for 5 min. The bands were visualized under ultraviolet (UV) light (handheld ultraviolet lamp 6-Watt, model 28191 B, Daigger Scientific Inc.) and isolated from the silica plates by scraping off the silica and transferring each band into a separate 1.5 mL Eppendorf tube. Next, the samples were suspended in 1 mL of 96% ethanol, mixed using a Vibromix, and centrifuged for 10 min at 20°C and 12000 × *g*. The obtained supernatants were separated from the remaining silica gel particles, transferred into clean Eppendorf tubes, and centrifuged again under the previously described conditions. All extraction steps were performed in the dark to avoid cis-trans photoisomerization and photodestruction because chlorophylls and carotenoids are light- and heat-sensitive [20, 21].

### Spectrophotometric assay

UV-Vis spectrophotometry (JENWAY 7315, Bibby Scientific Ltd.) was performed in the wavelength range of 350–750 nm and a spectral bandwidth of 1 nm was selected [14, 15]. The scanned samples contained 1 mL of the G1 and G2 leaf pigment extracts and 1 mL of each dissolved pigment band. The obtained spectra were used for subsequent investigations.

### Indication and quantitative evaluation of the pigments

Spectrophotometric data were processed using the GPS method for the qualitative and quantitative analysis of undefined mixtures, first described by Küpper et al. [13] and developed by Thrane et al. [15] using the R program. Calculations were performed using R version 3.6.1. of the RStudio interface version 1.2.5019 (R Core Team 2019) and R-functions provided by Thrane et al. [15] in the “nnls” library. Briefly, the background component spectra over wavelength range 350–750 nm [14], 390–710 nm, and 400–700 nm [15] were generated, and the absorption spectra were fitted using a non-negative least-squares approximation.

### Statistical analysis

#### Initial calculations

The compositions of the samples that had been separated by TLC were calculated using the Microsoft Excel (2019) software program. The pigment concentrations identified using the GPS method in every band were pooled, and each pigment proportion in the samples was obtained.

#### Mean values

Mean values of the number of indicated pigments and estimated concentrations in each wavelength range were calculated using SPSS ver. 26.

#### Variances

Variances in the concentrations of total pigments, chlorophylls and derivatives, and carotenoids in different wavelength ranges were calculated using the VAR.P function of the Microsoft Excel (2019) software program [22].

#### Levene’s test of variance homogeneity

Levene’s test [23] was performed in RStudio (R version 4.0.2) using the “leveneTest” function from the “car” package with an alfa level of 0.05 [24]. The variances in the results of the total pigment concentrations and concentrations of each pigment in each sample group and wavelength range, obtained before and after TLC analysis, were evaluated for homogeneity.

#### T-test

A two-sample t-test was used to assess whether the calculated total concentration, chlorophylls and derivatives, and carotenoids were significantly different depending on the storage conditions (G1 and G2) in each wavelength range.

Differences in these three concentrations, and each of the pigments calculated before and after TLC based on the different storage groups, were examined using a paired t-test [25].

Each test was performed in RStudio (R version 4.0.2) using the “t.test” function from the “psych” package.

#### Analysis of variance (ANOVA)

A one-way ANOVA was applied to each group to investigate whether a change in the wavelength range had a significant effect on the calculated total pigment, chlorophyll, and carotenoid content before and after TLC application [26, 27].

A two-way ANOVA was used to determine differences in calculated concentrations of each pigment between wavelength ranges, and between pigment concentrations in each Group for TLC separated and unseparated samples [28].

The calculations were performed in RStudio (R version 3.6.1) using “aov” [29] with an alpha level of 0.05 [30].

#### Interactive data visualization

Interactive visualization of the obtained data provides effective and efficient communication of the results [31]. All graphs were created using Python (version 3.7.0).

## Results

### GPS results of the unseparated samples

A change in the wavelength range resulted in varying indications and concentration estimations of the present pigments in the samples, for which the TLC step was omitted. The changes included the number of determined pigments, and their type and concentration (Table 1). The highest number of pigments was identified in the range of 400–700 nm, whereas the range of 350–750 nm failed to recognize any pigments and was therefore excluded from the further analysis.

**Table 1:** Concentrations of pigments in samples stored at different temperatures estimated using the GPS method in various wavelength ranges.

Pigment	Wavelength range (nm)	Sample size	20°C	-20°C
			Mean±SE (mg L^−1^)	Mean±SE (mg L^−1^)
**Allo**	350–750	3	0.00	0.00
	390–710	3	0.00	0.0106±0.0106
	400–700	3	0.00663±0.00509	0.00
**ββ.Car**	350–750	3	0.00	0.00
	390–710	3	0.00	0.00
	400–700	3	0.00	0.00
**C.Neo**	350–750	3	0.00	0.00
	390–710	3	0.00	0.00
	400–700	3	0.00	0.00
**Chl.a**	350–750	3	0.00	0.00
	390–710	3	0.00	0.00
	400–700	3	0.00	0.00
**Chl.b**	350–750	3	0.00	0.00
	390–710	3	0.163±0.0543	0.460±0.112
	400–700	3	0.0758±0.0122	0.189±0.0486
**Chl.c1**	350–750	3	0.00	0.00
	390–710	3	0.00	0.00
	400–700	3	0.00	0.00
**Chl.c2**	350–750	3	0.00	0.00
	390–710	3	0.00	0.00
	400–700	3	0.00	0.00
**Diadino**	350–750	3	0.00	0.00
	390–710	3	0.00	0.00
	400–700	3	0.120±0.0304	0.257±0.0564
**Diato**	350–750	3	0.00	0.00
	390–710	3	0.00	0.00
	400–700	3	0.00	0.00
**Dino**	350–750	3	0.00	0.00
	390–710	3	0.00	0.00
	400–700	3	0.00	0.00
**Echin**	350–750	3	0.00	0.00
	390–710	3	0.00	0.00
	400–700	3	0.00	0.00
**Fuco**	350–750	3	0.00	0.00
	390–710	3	0.00	0.00
	400–700	3	0.00	0.00
**Lut**	350–750	3	0.00	0.00
	390–710	3	0.00	0.00
	400–700	3	0.00	0.00
**Myxo**	350–750	3	0.00	0.00
	390–710	3	0.105±0.00662	0.165±0.0464
	400–700	3	0.00	0.00
**Peri**	350–750	3	0.00	0.00
	390–710	3	0.0261±0.0171	0.00
	400–700	3	0.147±0.0155	0.139±0.0682
**Phe.a**	350–750	3	0.00	0.00
	390–710	3	0.00	0.00
	400–700	3	0.0127±0.0127	0.00
**Phe.b**	350–750	3	0.00	0.00
	390–710	3	0.00	0.00
	400–700	3	0.00	0.00
**Viola**	350–750	3	0.00	0.00
	390–710	3	0.00	0.00
	400–700	3	0.00	0.00

Note: Allo = Alloxanthin; ββ.car = *β*,*β*-Carotene; C.neo = 9’-*cis*-Neoxanthin; Cantha = *trans*-Canthaxanthin; Chl.a = Chlorophyll *a*; Chl.b = Chlorophyll *b*; Chl.c1 = Chlorophyll *c*_2_; Chl.c2 = Chlorophyll *c*_2_; Diadino = *trans*-Diadinoxanthin; Diato = Diatoxanthin; Dino = Dinoxanthin; Echin = *trans*-Echinenone; Fuco = Fucoxanthin; Lut = Lutein; Myxo = Myxoxanthophyll; Peri = Peridinin; Phe.a = Pheophytin *a*; Phe.b = Pheophytin *b*; Viola = Violaxanthin.

Variances in the three calculated concentrations (total concentration, chlorophylls and derivatives, and carotenoids) (Table 2) determined in every range for each sample group (Table 3) were found to be homogenous (p > 0.05) using Levene’s test (Table 6). The one-way ANOVA analysis showed no statistically significant difference (p > 0.05) in the total concentration means in each wavelength range for the G1 and G2 samples, except for the total carotenoid content in samples stored at room temperature that were different (Table 4).

**Table 2:** Calculated concentrations of total pigment in samples stored at different temperatures estimated using the GPS method in various wavelength ranges.

Pigment	Wavelength range (nm)	Sample size	20°C	-20°C
			Mean±SE (mg L^−1^)	Mean±SE (mg L^−1^)
**Total**	350–750	3	0.00	0.00
	390–710	3	0.636±0.161	0.295±0.0640
	400–700	3	0.611±0.177	0.361±0.0106
**Chlorophylls and derivatives**	350–750	3	0.00	0.00
	390–710	3	0.460±0.112	0.163±0.0543
	400–700	3	0.189±0.0486	0.0885±0.0249
**Carotenoids**	350–750	3	0.00	0.00
	390–710	3	0.176±0.0489	0.131±0.0106
	400–700	3	0.422±0.129	0.273±0.0181

**Table 3:** Variances in the estimated total pigment concentrations in samples stored at different temperatures according to different wavelength ranges.

Pigment	Wavelength ranges (nm)	GPS		GPS with TLC	
		20°C	-20°C	20°C	-20°C
**Total (mg^2^L)**	All wavelength ranges	-	-	10.7	1.13
	350–750 and 390–710	-	-	12.8	1.25
	390–710 and 400–700	0.0572	< 0.0100	6.91	0.173
	350–750 and 400–700	-	-	12.4	1.52
**Chlorophylls and derivatives (mg^2^L)**	All wavelength ranges	-	-	3.54	3.89
	350–750 and 390–710	-	-	5.38	4.27
	390–710 and 400–700	0.0332	< 0.0100	1.89	0.476
	350–750 and 400–700	-	-	4.50	4.59
**Carotenoids (mg^2^L)**	All wavelength ranges	-	-	2.10	0.881
	350–750 and 390–710	-	-	2.29	1.08
	390–710 and 400–700	0.0342	< 0.0100	1.90	0.345
	350–750 and 400–700	-	-	2.10	1.11

**Table 4:** P-values of the one-way ANOVA for differences in estimated total pigment concentrations between all wavelength ranges according to the storage temperature.

Pigment	GPS		GPS with TLC	
	20°C	-20°C	20°C	-20°C
**Total**	0.363	0.922	0.969	0.0768
**Chlorophylls and derivatives**	0.279	0.0903	0.923	0.215
**Carotenoids**	< 0.0100	0.150	0.972	0.589

The estimated concentrations of each identified pigment in each group sample showed homogenous variances (Table 3) in all wavelength ranges (Tables 5 and 6). However, the two-way ANOVA showed that the mean contents of the majority of the compounds in each group were statistically different between wavelength ranges, with alloxanthin and pheophytin *a* being identical (Table 7).

**Table 5:** P-values of Levene’s test for homogeneity of variances in the estimated pigment concentrations (Allo to Dino) in all wavelength ranges according to the storage temperature.

Pigment	GPS		GPS with TLC	
	20°C	-20°C	20°C	-20°C
**Allo**	0.242	0.374	0.998	0.602
**ββ.car**	-	-	0.909	0.606
**C.neo**	-	-	0.116	0.541
**Cantha**	-	-	-	-
**Chl.a**	-	-	0.478	0.709
**Chl.b**	0.393	0.557	0.415	0.956
**Chl.c1**	-	-	0.817	0.740
**Chl.c2**	-	-	0.541	0.411
**Diadino**	0.247	0.117	0.977	0.580
**Diato**	-	-	0.891	0.831
**Dino**	-	-	0.422	0.347

Note: Allo = Alloxanthin; ββ.car = *β*,*β*-Carotene; C.neo = 9’-*cis*-Neoxanthin; Cantha = *trans*-Canthaxanthin; Chl.a = Chlorophyll *a*; Chl.b = Chlorophyll *b*; Chl.c1 = Chlorophyll *c*_2_; Chl.c2 = Chlorophyll *c*_2_; Diadino = *trans*-Diadinoxanthin; Diato = Diatoxanthin; Dino = Dinoxanthin.

**Table 6:** P-values of Levene’s test for homogeneity of variances in the estimated pigment concentrations (Echin to Viola) and total concentrations in all wavelength ranges according to the storage temperature.

Pigment	GPS		GPS with TLC	
	20°C	-20°C	20°C	-20°C
**Echin**	-	-	0.552	-
**Fuco**	-	-	0.995	-
**Lut**	-	-	0.381	0.422
**Myxo**	0.245	0.136	0.458	0.504
**Peri**	0.878	0.146	0.490	0.228
**Phe.a**	0.374	-	0.665	0.691
**Phe.b**	-	-	0.814	0.619
**Viola**	-	-	0.917	0.428
**Total**	0.891	0.306	0.910	0.318
**Chlorophylls and derivatives**	0.557	0.564	0.801	0.336
**Carotenoids**	0.438	0.522	0.989	0.698

Note: Echin = *trans*-Echinenone; Fuco = Fucoxanthin; Lut = Lutein; Myxo = Myxoxanthophyll; Peri = Peridinin; Phe.a = Pheophytin *a*; Phe.b = Pheophytin *b*; Viola = Violaxanthin.

**Table 7:** P-values of two-way ANOVA for differences in estimated pigment concentrations between wavelength ranges and storage temperatures respectively.

Pigment	Wavelength range		Storage temperature	
	GPS	GPS with TLC	GPS	GPS with TLC
**Allo**	0.744	0.667	0.744	0.245
**ββ.car**	-	0.712	-	0.384
**C.neo**	-	**-**	**-**	**-**
**Cantha**	-	0.611	-	< 0.0100
**Chl.a**	-	0.383	-	0.0757
**Chl.b**	0.0281	0.792	0.0155	0.471
**Chl.c1**	-	0.735	-	0.154
**Chl.c2**	-	0.390	-	0.550
**Diadino**	< 0.0100	0.688	0.0652	0.823
**Diato**	-	0.615	-	< 0.0100
**Dino**	-	0.0777	-	0.9955
**Echin**	-	0.536	-	0.219
**Fuco**	-	0.995	-	0.109
**Lut**	-	0.339	-	0.459
**Myxo**	< 0.0100	0.111	0.235	0.290
**Peri**	0.00687	0.256	0.657	0.330
**Phe.a**	0.347	0.108	0.347	0.0821
**Phe.b**	-	0.578	-	0.140
**Viola**	-	0.465	-	0.737

Note: Allo = Alloxanthin; ββ.car = *β*,*β*-Carotene; C.neo = 9’-*cis*-Neoxanthin; Cantha = *trans*-Canthaxanthin; Chl.a = Chlorophyll *a*; Chl.b = Chlorophyll *b*; Chl.c1 = Chlorophyll *c*_2_; Chl.c2 = Chlorophyll *c*_2_; Diadino = *trans*-Diadinoxanthin; Diato = Diatoxanthin; Dino = Dinoxanthin; Echin = *trans*-Echinenone; Fuco = Fucoxanthin; Lut = Lutein; Myxo = Myxoxanthophyll; Peri = Peridinin; Phe.a = Pheophytin *a*; Phe.b = Pheophytin *b*; Viola = Violaxanthin.

### GPS results of the separated samples

The combination of the GPS method with TLC indicated the presence of pigments in all wavelength ranges (Tables 8 and 9). The variances in the total pigment, chlorophyll and derivatives, and carotenoid concentrations (Table 10) in all wavelength ranges were homogenous for the G1 and G2 samples (Table 6). When the concentrations were paired, the lowest variances, and thus differences between results, were observed in the 390–710 nm and 400–700 nm ranges for each group and all categories (Table 3). Variances in every pigment concentrations in each wavelength range based on the groups (Table 3) were found to be homogenous (Tables 5 and 6). The two-way ANOVA showed no statistically significant difference in the mean concentrations of each pigment calculated in all ranges and for each group (Table 7).

**Table 8:** Concentrations of pigments (Allo to Fuco) in samples stored at different temperatures estimated using the GPS method with TLC in various wavelength ranges.

Pigmenth	Wavelength range (nm)	Sample size	20°C	-20°C
			Mean±SE (mg L^−1^)	Mean±SE (mg L^−1^)
**Allo**	350–750	3	0.560±0.528	0.877±0.272
	390–710	3	0.710±0.572	1.57±0.823
	400–700	3	0.921±0.525	1.57±0.782
**ββ.Car**	350–750	3	0.133±0.0665	0.00
	390–710	3	0.0619±0.0357	0.313±0.313
	400–700	3	0.0650±0.0411	0.390±0.367
**C.Neo**	350–750	3	0.00	0.0153±0.00815
	390–710	3	0.00382±0.00382	0.0154±0.00649
	400–700	3	0.00168±0.00108	0.0214±0.00243
**Chl.a**	350–750	3	0.0471±0.0258	0.0120±0.0106
	390–710	3	0.0164±0.00891	0.00213±0.00213
	400–700	3	0.0276±0.0149	0.0102±0.0101
**Chl.b**	350–750	3	0.0276±0.0149	0.954±0.954
	390–710	3	0.544±0.224	0.841±0.817
	400–700	3	0.971±0.443	0.837±0.568
**Chl.c1**	350–750	3	0.00161±0.00161	0.178±0.178
	390–710	3	0.00667±0.00667	0.0599±0.0599
	400–700	3	0.00205±0.00197	0.0719±0.0709
**Chl.c2**	350–750	3	0.00903±0.00233	0.00360±0.00995
	390–710	3	0.00643±0.00116	0.00290±0.00763
	400–700	3	0.00735±0.00103	0.0362±0.0322
**Diadino**	350–750	3	0.514±0.408	0.0395±0.0127
	390–710	3	0.556±0.545	0.795±0.700
	400–700	3	0.458±0.419	1.01±0.893
**Diato**	350–750	3	0.583±0.207	4.88±1.06
	390–710	3	0.304±0.163	3.61±1.70
	400–700	3	0.292±0.147	3.10±1.65
**Dino**	350–750	3	0.0813±0.0813	0.0808±0.0405
	390–710	3	0.00	0.00
	400–700	3	0.00	0.00
**Echin**	350–750	3	0.00	0.00
	390–710	3	0.00999±0.00999	0.00
	400–700	3	0.00252±0.00252	0.00
**Fuco**	350–750	3	0.0108±0.0108	0.00
	390–710	3	0.0109±0.0109	0.00
	400–700	3	0.00951±0.00951	0.00

Note: Allo = Alloxanthin; ββ.car = *β*,*β*-Carotene; C.neo = 9’-*cis*-Neoxanthin; Cantha = *trans*-Canthaxanthin; Chl.a = Chlorophyll *a*; Chl.b = Chlorophyll *b*; Chl.c1 = Chlorophyll *c*_2_; Chl.c2 = Chlorophyll *c*_2_; Diadino = *trans*-Diadinoxanthin; Diato = Diatoxanthin; Dino = Dinoxanthin; Echin = *trans*-Echinenone; Fuco = Fucoxanthin.

**Table 9:** Concentrations of pigments (Lut to Viola) in samples stored at different temperatures estimated using the GPS method with TLC in various wavelength ranges.

Pigment	Wavelength range (nm)	Sample size	20°C	-20°C
			Mean±SE (mg L^−1^)	Mean±SE (mg L^−1^)
**Lut**	350–750	3	0.00	0.00
	390–710	3	0.0427±0.0365	0.00
	400–700	3	0.0657±0.0408	0.473±0.473
**Myxo**	350–750	3	0.00401±0.00397	0.0124±0.0111
	390–710	3	0.179±0.142	0.101±0.0852
	400–700	3	0.383±0.196	0.149±0.0961
**Peri**	350–750	3	0.0326±0.0106	0.0104±0.00669
	390–710	3	0.0108±0.00429	0.00197±0.00197
	400–700	3	0.00427±0.00427	0.107±0.0694
**Phe.a**	350–750	3	1.93± 1.92	4.27±0.910
	390–710	3	0.698±0.642	2.01±0.607
	400–700	3	0.537±0.483	1.43±0.460
**Phe.b**	350–750	3	0.0125±0.00676	0.0344±0.0107
	390–710	3	0.0206±0.0151	0.352±0.312
	400–700	3	0.0239±0.0104	0.348±0.295
**Viola**	350–750	3	0.0574±0.0300	0.00121±0.00121
	390–710	3	0.0476±0.0259	0.104±0.104
	400–700	3	0.0428±0.0215	0.00126±0.00769

Note: Lut = Lutein; Myxo = Myxoxanthophyll; Peri = Peridinin; Phe.a = Pheophytin *a*; Phe.b = Pheophytin *b*; Viola = Violaxanthin.

**Table 10:** Calculated concentrations of total pigment in samples stored at different temperatures estimated using the GPS method with TLC in various wavelength ranges.

Pigment	Wavelength range (nm)	Sample size	20°C	-20°C
			Mean± SE (mg L^−1^)	Mean± SE (mg L^−1^)
**Total**	350–750	3	4.00±3.02	11.4±0.769
	390–710	3	3.21±1.87	9.78±0.160
	400–700	3	3.81±1.82	9.56±0.326
**Chlorophylls and derivatives**	350–750	3	2.03±1.90	5.45±1.644
	390–710	3	1.29±0.850	3.27±0.606
	400–700	3	1.57±0.910	2.73±0.191
**Carotenoids**	350–750	3	1.98±1.12	5.92±0.887
	390–710	3	1.92±1.02	6.51±0.455
	400–700	3	2.24±0.911	6.83±0.337

### Differences in concentrations due to storage conditions

The results of the two-sample t-test showed that the G2 samples not separated by TLC in the 400–700 nm range contained total pigment, chlorophyll, and carotenoid concentrations that were not significantly greater than those in the G1 samples. In the 390–710 nm range, a significantly greater concentration in the G2 samples was only calculated for chlorophyll (Table 11).

**Table 11:** P-values of the two-sample t-test for differences in estimated total pigment concentrations between storage temperatures.

Pigment	GPS		GPS with TLC		
	390–710 nm	400–700 nm	350–750 nm	390–710 nm	400–700 nm
**Total**	0.0597	0.115	0.0387	0.0125	0.0180
**Chlorophylls and derivatives**	0.0376	0.0691	0.122	0.0654	0.125
**Carotenoids**	0.212	0.159	0.0254	< 0.0100	< 0.0100

The two-way ANOVA concluded that none of the mean pigment concentrations belonging to the carotene group or pheophytin *a* were statistically different in the G2 samples compared to the G1 samples for both wavelength ranges of the TLC unseparated samples. A significant difference was observed only between chlorophyll *b* concentrations. Each pigment concentration calculated after TLC in all wavelength ranges was the same for the chlorophylls and derivatives groups. For carotenes, a significant difference was observed in neoxanthin and diatoxanthin concentrations (Table 7).

### Comparison between unseparated and separated samples

**Fig 2:**
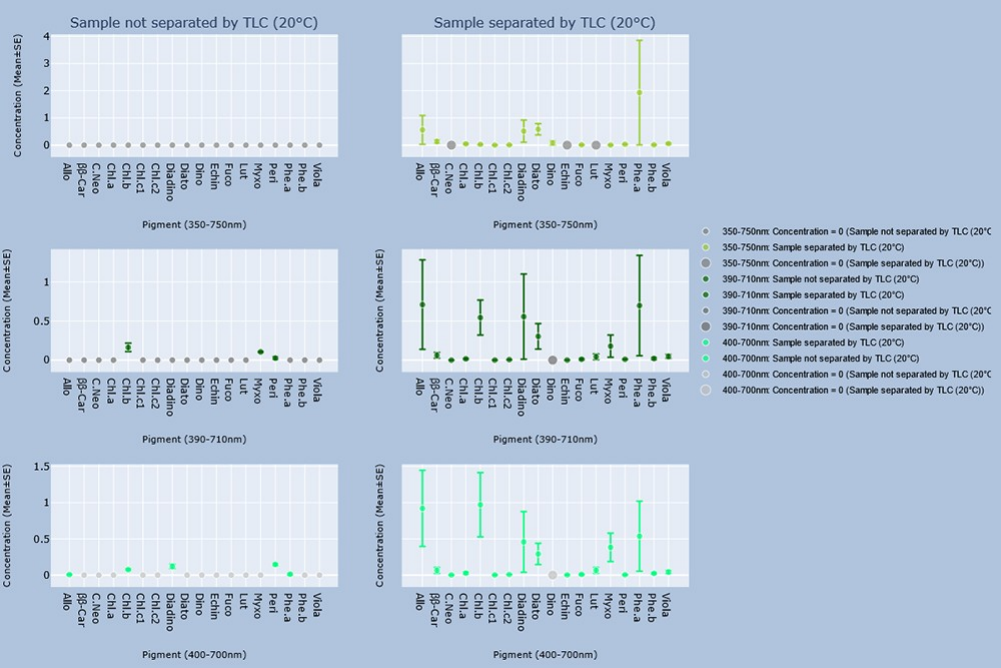
Concentrations of pigments estimated by the GPS method, and GPS method with TLC in samples stored at 20°C. Interactive visualization of the data has been published online at https://www.ebi.ac.uk/biostudies/studies/S-BSST642. Allo = Alloxanthin; ββ-Car = β,β-Carotene; C.Neo = 9’-*cis*-Neoxanthin; Cantha = *trans*-Canthaxanthin; Chl.a = Chlorophyll a; Chl.b = Chlorophyll b; Chl.c1 = Chlorophyll c_2_; Chl.c2 = Chlorophyll c_2_; Diadino = *trans*-Diadinoxanthin; Diato = Diatoxanthin; Dino = Dinoxanthin; Echin = *trans*-Echinenone; Fuco = Fucoxanthin; Lut = Lutein; Myxo = Myxoxanthophyll; Peri = Peridinin; Phe.a = Pheophytin a; Phe.b = Pheophytin b; Viola = Violaxanthin.

**Fig 3:**
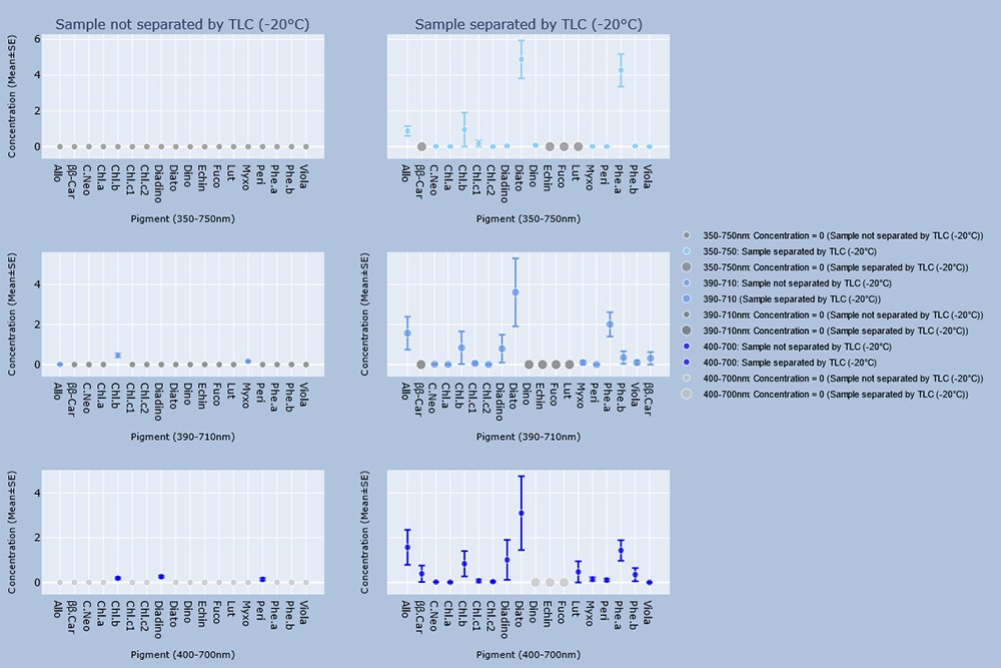
Concentrations of pigments estimated by the GPS method, and GPS method with TLC in samples stored at -20°C. Interactive visualization of the data has been published online at https://www.ebi.ac.uk/biostudies/studies/S-BSST642. Allo = Alloxanthin; ββ-Car = β,β-Carotene; C.Neo = 9’-*cis*-Neoxanthin; Cantha = *trans*-Canthaxanthin; Chl.a = Chlorophyll a; Chl.b = Chlorophyll b; Chl.c1 = Chlorophyll c_2_; Chl.c2 = Chlorophyll c_2_; Diadino = *trans*-Diadinoxanthin; Diato = Diatoxanthin; Dino = Dinoxanthin; Echin = *trans*-Echinenone; Fuco = Fucoxanthin; Lut = Lutein; Myxo = Myxoxanthophyll; Peri = Peridinin; Phe.a = Pheophytin a; Phe.b = Pheophytin b; Viola = Violaxanthin.

**Fig 4:**
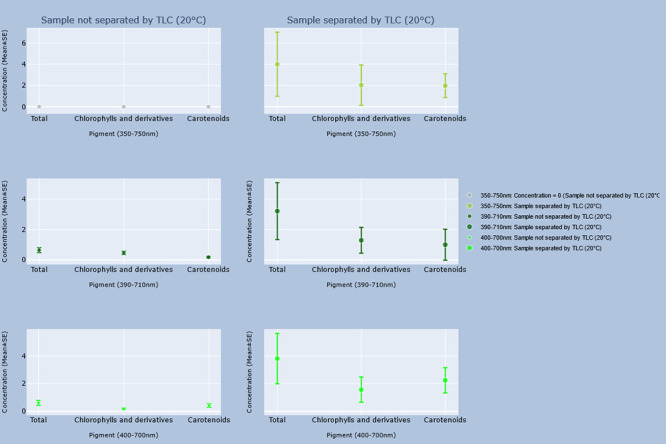
Concentrations of total pigment estimated by the GPS method, and GPS method with TLC in samples stored at 20°C. Interactive visualization of the data has been published online at https://www.ebi.ac.uk/biostudies/studies/S-BSST642. Allo = Alloxanthin; ββ-Car = β,β-Carotene; C.Neo = 9’-*cis*-Neoxanthin; Cantha = *trans*-Canthaxanthin; Chl.a = Chlorophyll a; Chl.b = Chlorophyll b; Chl.c1 = Chlorophyll c_2_; Chl.c2 = Chlorophyll c_2_; Diadino = *trans*-Diadinoxanthin; Diato = Diatoxanthin; Dino = Dinoxanthin; Echin = *trans*-Echinenone; Fuco = Fucoxanthin; Lut = Lutein; Myxo = Myxoxanthophyll; Peri = Peridinin; Phe.a = Pheophytin a; Phe.b = Pheophytin b; Viola = Violaxanthin.

**Fig 5:**
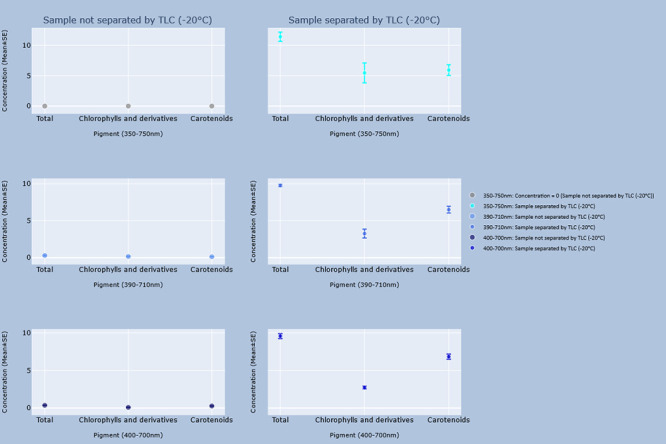
Concentrations of total pigment estimated by the GPS method, and GPS method with TLC in samples stored at -20°C. Interactive visualization of the data has been published online at https://www.ebi.ac.uk/biostudies/studies/S-BSST642. Allo = Alloxanthin; ββ-Car = β,β-Carotene; C.Neo = 9’-*cis*-Neoxanthin; Cantha = *trans*-Canthaxanthin; Chl.a = Chlorophyll a; Chl.b = Chlorophyll b; Chl.c1 = Chlorophyll c_2_; Chl.c2 = Chlorophyll c_2_; Diadino = *trans*-Diadinoxanthin; Diato = Diatoxanthin; Dino = Dinoxanthin; Echin = *trans*-Echinenone; Fuco = Fucoxanthin; Lut = Lutein; Myxo = Myxoxanthophyll; Peri = Peridinin; Phe.a = Pheophytin a; Phe.b = Pheophytin b; Viola = Violaxanthin.

Differences between the concentrations of every identified pigment and total concentrations when TLC was or was not performed for each group are shown in the graphs in Figs 2–5. Every pigment identified without TLC application was identified in the samples with TLC, the concentrations of which were statistically identical (Table 12). However, the calculated concentrations of total pigment, chlorophyll and derivatives, and carotenoid concentrations in the 390–710 nm and 400–700 nm ranges from the spectrophotometric results of unseparated samples were significantly lower (p < 0.05) than those of the separated samples (Table 13).

**Table 12:** P-values of the paired t-test for differences in estimated pigment concentrations at different temperatures before and after TLC was applied to the GPS method.

Pigment	20°C		-20°C	
	390–710 nm	400–700 nm	390–710 nm	400–700 nm
**Allo**	-	0.226	0.201	-
**Chl.b**	0.222	0.185	0.722	0.403
**Diadino**	-	0.497	-	0.507
**Myxo**	0.651	-	0.668	-
**Peri**	0.459	0.2256	-	0.829
**Phe.a**	-	0.397	-	-

Note: Allo = Alloxanthin; Chl.b = Chlorophyll *b*; Diadino = *trans*-Diadinoxanthin; Myxo = Myxoxanthophyll; Peri = Peridinin; Phe.a = Pheophytin *a*.

**Table 13:** P-values of the paired t-test for differences in estimated total pigment concentrations at different temperatures before and after TLC was applied to the GPS method.

Pigment	20°C	-20°C
**Total**	0.0209	< 0.0100
**Chlorophylls and derivatives**	0.0342	< 0.0100
**Carotenoids**	0.0114	< 0.0100

## Discussion

### Influence of wavelength ranges in spectrophotometry on the GPS

The study showed a weakness of the GPS method that was not mentioned by Thrane et al. [15]. The choice of wavelength range in which the spectrophotometry was conducted significantly influenced the estimated concentrations of most pigments in the unseparated samples. Furthermore, in the wavelength range of 350–750 nm, no pigments were detected in the unseparated samples. In the spectrophotometric assay, a large absorption peak was observed in the UV region below 370 nm, possibly due to the phenolic structures present in the pigment extracts [32, 33], which might have influenced spectra fitting by non-negative least squares [34, 35], a crucial part of the GPS method, as Thrane et al. [15] recorded spectral scans only between 400 and 700 nm. The addition of TLC ensured the separation of phenolic compounds from the present pigments [36], thereby decreasing the influence of other compounds. When TLC was applied, the estimated concentrations of each pigment and the calculated total concentrations were statistically identical in all investigated wavelength ranges. Therefore, combining TLC with the GPS method overcame the recognized weaknesses by providing results that were less prone to being significantly different depending on the wavelength range choice. This advantage becomes crucial when a spectrophotometer with an ultraviolet region is not available. Moreover, it indicates consistency in the estimated pigment content and concentrations in the samples when the GPS method was combined with TLC, which could not be concluded from the results when this step was omitted.

### Differences in estimated pigment concentrations before and after TLC

The application of TLC resulted in the separation of major and minor chlorophyll and carotenoid components, leading to a reduced overlap of absorption peaks in the blue-green region during the spectrophotometric assay [20, 37–39]. Consequently, minor pigment component absorption peaks were detected using a UV-Vis spectrophotometer. Hence, the results showed an increased variety of pigments present in higher plant leaves, as described in the literature [40]. The total pigment concentrations were estimated to be significantly higher in the samples separated by TLC due to their increased number detected, which led to a change in the variance in the results compared to the unseparated samples. The concentrations of each pigment before and after TLC were statistically identical, proving that the size of pigment recoveries by TLC [41] had a minor influence on the obtained results. Therefore, adding TLC enhanced the ability of the GPS method to indicate the present pigments and estimate their concentrations.

### Behavior of samples under different conditions

Changes in the 390–710 nm range between the pigment composition of both unseparated groups were found only for chlorophyll b, and hence total chlorophylls and derivatives. However, this result is highly improbable, as chlorophylls are stable at both room temperature and in the freezer [42, 43]. When TLC was added, the estimated total pigment concentrations of chlorophylls and derivatives, and each pigment belonging to this group remained, according to the GPS results, the same regardless of the chosen range. A decrease in total carotenoids and total pigment concentration was observed in the samples stored at room temperature, which is in good agreement with the literature [44–46]. This was not concluded from the results of the unseparated samples. Therefore, conducting a spectrophotometric assay of TLC pigment bands allowed for the recognition of patterns regarding pigment concentration changes due to storage temperature.

### Choice of the wavelength range

The wavelength range of 350–750 nm failed to calculate the pigment concentration in the unseparated samples, whereas 390–710 nm indicated an improbable change in chlorophyll content due to the storage temperature of *A*. *hippocastanum* autumn leaves. In both unseparated and separated by TLC samples, the greatest number of pigments was shown for the wavelength range of 400–700 nm. The smallest variance in the concentrations of separated samples was recorded in the paired results at 390–710 nm and 400–700 nm. Due to a fairly small sample size, the calculated standard errors for pigment and total concentrations was relatively large [47]. However, the smallest standard errors overall were recorded in the range 400–700 nm for the TLC separated samples. Therefore, the present study provided evidence to suggest that a wavelength range of 400–700 nm is optimal for the GPS method, which has not been previously shown for this method [14, 15].

### Costs of the method

The addition of the TLC step to the GPS method did not introduce high additional costs [48]. Hence, the described method aligns well with the idea of an easy and inexpensive procedure [14, 15].

### Indicated pigments and future perspectives

The GPS method described by Thrane et al. [15] was reported as a successful alternative to the HPLC method. However, the results from the present study demonstrated strong evidence that the addition of the TLC step to the GPS method provided more reliable results in the investigated aspects. Hence, this new method is expected to be an even more successful alternative to HPLC. However, the study revealed a possible weakness of the GPS method whether combined with TLC or not. In both cases, some of the indicated pigments were not typical constituents of terrestrial plants [49–51], although such cases were sometimes reported [52]. Therefore, one direction of future research should involve comparing identified pigments in *A*. *hippocastanum* leaves using the GPS method and TLC with the results obtained from another pigment identifying and quantifying method such as HPLC.

Apart from *A*. *hippocastanum*, there are several other trees, such as *Betula pendula* and *Acer pseudoplatanus L*., which could be potential natural pigment sources. Therefore, another direction of further study should involve these species. As the food industry is seeking stable, non-toxic colorants, the obtained pigments could be tested for eligibility in the future and, if successful, could potentially revolutionize the market.

## Conclusions

The present study conducted TLC prior to spectrophotometric analysis to improve the ability of the GPS method to identify the pigments present. A change in the wavelength range over which the absorption spectra were generated had an insignificant effect on the determined pigments and their number when the components were separated. The use of the three wavelength ranges for the data obtained from the unseparated samples led to differences in the indicated pigments and their estimated concentrations. The concentrations calculated from the absorption spectra within the wavelength range of 400–700 nm were the most representative among the sample compounds for both approaches to the GPS method.

## Supporting information

S1 File (TXT)
