# Correction: Enhanced pigment content estimation using the Gauss-peak spectra method with thin-layer chromatography for a novel source of natural colorants

**DOI:** 10.1371/journal.pone.0307583

**Published:** 2024-07-17

**Authors:** Natalia Paulina Twardowska

In Fig 1, the storage temperatures indicated are incorrectly labelled. It should have been 20°C and -20°C. Please see the correct Fig 1 here.

**Fig 1:**
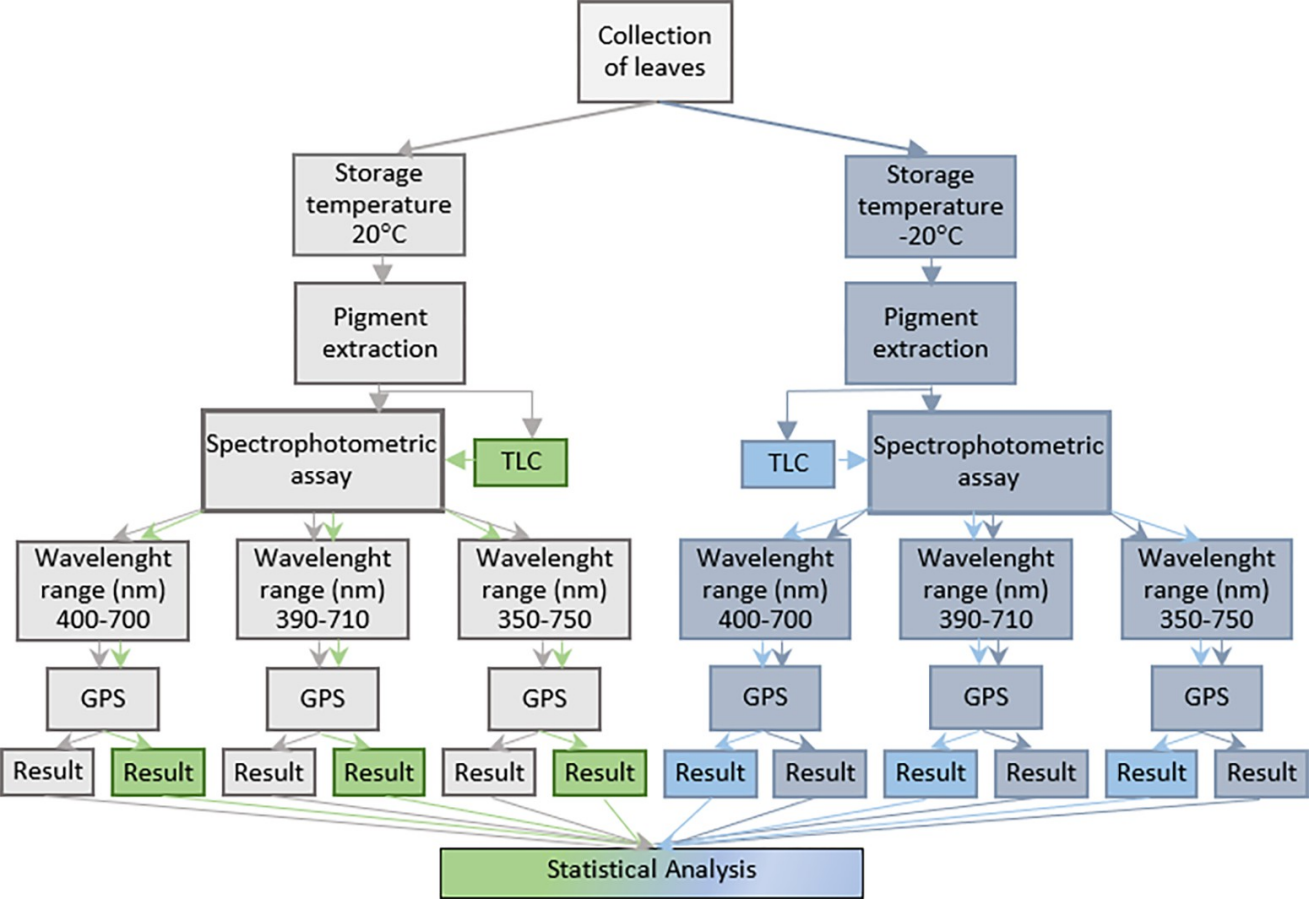
Flow chart of the pigment quantification processes.
